# Next Generation Risk Assessment of the Anti-Androgen Flutamide Including the Contribution of Its Active Metabolite Hydroxyflutamide

**DOI:** 10.3389/ftox.2022.881235

**Published:** 2022-06-02

**Authors:** Tessa C.A. van Tongeren, Paul L. Carmichael, Ivonne M.C.M. Rietjens, Hequn Li

**Affiliations:** ^1^ Division of Toxicology, Wageningen University and Research, Wageningen, Netherlands; ^2^ Unilever Safety and Environmental Assurance Centre, Sharnbrook, United Kingdom

**Keywords:** risk assessment, 3R compliant method, PBK modelling, anti-androgens, *in vitro*/in silico approaches

## Abstract

In next generation risk assessment (NGRA), non-animal approaches are used to quantify the chemical concentrations required to trigger bioactivity responses, in order to assure safe levels of human exposure. A limitation of many *in vitro* bioactivity assays, which are used in an NGRA context as new approach methodologies (NAMs), is that toxicokinetics, including biotransformation, are not adequately captured. The present study aimed to include, as a proof of principle, the bioactivity of the metabolite hydroxyflutamide (HF) in an NGRA approach to evaluate the safety of the anti-androgen flutamide (FLU), using the AR-CALUX assay to derive the NAM point of departure (PoD). The NGRA approach applied also included PBK modelling-facilitated quantitative *in vitro* to *in vivo* extrapolation (QIVIVE). The PBK model describing FLU and HF kinetics in humans was developed using GastroPlus™ and validated against human pharmacokinetic data. PBK model-facilitated QIVIVE was performed to translate the *in vitro* AR-CALUX derived concentration-response data to a corresponding *in vivo* dose-response curve for the anti-androgenicity of FLU, excluding and including the activity of HF (-HF and +HF, respectively). The *in vivo* benchmark dose 5% lower confidence limits (BMDL_05_) derived from the predicted *in vivo* dose-response curves for FLU, revealed a 440-fold lower BMDL_05_ when taking the bioactivity of HF into account. Subsequent comparison of the predicted BMDL_05_ values to the human therapeutic doses and historical animal derived PoDs, revealed that PBK modelling-facilitated QIVIVE that includes the bioactivity of the active metabolite is protective and provides a more appropriate PoD to assure human safety *via* NGRA, whereas excluding this would potentially result in an underestimation of the risk of FLU exposure in humans.

## 1 Introduction

Many toxicologists have long aimed to replace, reduce, and refine (3Rs) the use of animals for experimentation (Russell and Burch, 1959) in assuring safe levels of human exposure to chemicals. The use of new approach methodologies (NAMs) in next generation risk assessment (NGRA) has become a solution to this goal (US Environmental Protection Agency, 2018). In this context, *in vitro* cell-based assays have been developed and used to quantify toxicodynamic responses of chemicals to predict the potential corresponding *in vivo* responses (National Research Council, 2007; Carmichael et al., 2009) or to define a safe (protective) level of exposure to a chemical agent or ingredient (Baltazar et al., 2020). Ongoing developments seek to translate the *in vitro* responses to the corresponding *in vivo* responses in humans or to determine the ideal battery of NAMs to define safe exposure levels in humans, without aiming to predict levels of expected animal pathology. One particular limitation of simple *in vitro* cell-based systems, however, is that they are rarely able to replicate the toxicokinetics of a compound, as seen in the *in vivo* situation and therefore the exact pattern of exposure at the biological target site (Coecke et al., 2006; OECD 2006; Mazzoline et al., 2009; Hartung 2018). Metabolic biotransformation, for instance, can result in bioactivation or detoxication of compounds and thus change their potency at their biological target in the human body (Coecke et al., 2006; OECD 2006; Gu and Manautou 2012).

**FIGURE 1:**
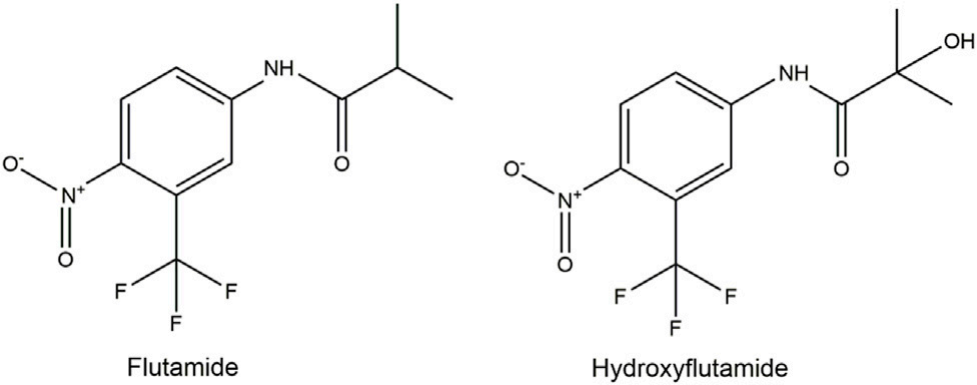
Structure formulas of flutamide and hydroxyflutamide.

The present study aimed to include, as a proof of principle, the bioactivity of the metabolite hydroxyflutamide (HF, Figure 1) in an NGRA approach to evaluate the safety of the pharmaceutical anti-androgen flutamide (FLU, Figure 1) based on a point of departure (PoD) derived from the validated *in vitro* androgen receptor (AR)-CALUX assay (Sonneveld et al., 2005; van der Burg et al., 2010). The approach applied included physiologically based kinetic (PBK) modelling-facilitated quantitative *in vitro* to *in vivo* extrapolation (QIVIVE). PBK modelling enables the in silico simulation of the absorption, distribution, metabolism and excretion of chemicals in the human body based on physiological, physicochemical, and kinetic parameters. This allows the prediction and subsequent interpretation of concentrations of a parent compound and its relevant metabolites for a certain time-point, route of administration, and dose in specific target organs. Thus, PBK modelling is a useful tool in the translation of *in vitro* concentration-response data to *in vivo* dose-response data (Rietjens et al., 2011; Zhang M. et al., 2018; Li et al., 2021).

In this study, FLU was selected as model compound. FLU is a nonsteroidal anti-androgen used in the treatment for prostate cancer or hirsutism and metabolised to its more anti-androgenic active metabolite HF (Figure 1) (Schultz et al., 1988; Radwanski et al., 1989; McLeod, 1993; Shet et al., 1997; Calaf et al., 2017). FLU is selected as model compound since it is a well characterized chemical with high human and historical animal data availability to validate the approach. This approach however may also be used to evaluate not just pharmaceutical agents but also other chemicals such as cosmetics, pesticides, and other environmental agents. The hydroxylation of FLU occurs predominantly in the liver and is catalysed by cytochrome P450 (CYP) enzymes. CYP1A2 is the major enzyme responsible for the conversion but CYP1A1, CYP1B1, and CYP3A4 are also involved (Shet et al., 1997; Tevell et al., 2006; Kang et al., 2008). Besides its conversion to HF, FLU is hepatically converted to other metabolites, but no anti-androgenic activity has been reported for these compounds (Shet et al., 1997; Tevell et al., 2006; Kang et al., 2008). Upon its formation, HF is conjugated by hepatic enzymes and excreted in urine (Shet et al., 1997; Tevell et al., 2006; Kostrubsky et al., 2007; Kang et al., 2008). The anti-androgenicity of FLU can be quantified in the AR-CALUX assay (Sonneveld et al., 2005; van der Burg et al., 2010). Given the bioactivity of HF, it is likely that the FLU-dependent anti-androgenic response in humans is to be incompletely predicted if based solely on the results of the *in vitro* AR-CALUX assay, as this metabolism does not occur under the *in vitro* assay conditions. Therefore, the contribution of the bioactivity of HF was included in the PBK modelling-facilitated QIVIVE of the anti-androgenic activity of FLU based on the *in vitro* AR-CALUX assay in order to derive a more *in vivo* relevant PoD. The Michaelis-Menten kinetic parameters for the hydroxylation of FLU to HF and the hepatic clearance (CL_int_) of FLU were obtained following incubations with microsomes from human liver. The CL_int_ of HF was determined following incubation with the human hepatoma HepaRG cell line (Gripon et al., 2002; Aninat et al., 2006). The PBK model describing FLU and HF kinetics in humans was then developed using GastroPlus™ and validated against human *in vivo* pharmacokinetic data. PBK modelling-facilitated QIVIVE was performed to translate the *in vitro* AR-CALUX derived concentration-response curve for FLU to the corresponding dose-response curves for the anti-androgenicity of FLU, either excluding or including the anti-androgenic activity of HF (-HF and +HF, respectively). Benchmark dose (BMD) analysis of the derived dose-response curves was performed to obtain the *in vivo* benchmark dose 5% lower confidence limits (BMDL_05_) as PoDs for comparison to human therapeutic doses and historical animal derived PoDs of FLU (Schellhammer et al., 1997; Calaf et al., 2017; Zacharia, 2017) to evaluate the use of the NGRA approach to define safe levels of human exposure to FLU.

## 2 Material and Methods

### 2.1 Materials

DHT (CAS no. 521–18-6), FLU (CAS no. 13311–84-7), HF (CAS no. 52806-53-8), tributyltin acetate (TBTa, Cas no. 56-36-0), reduced nicotinamide adenine dinucleotide phosphate (NADPH), alamethicin, magnesium chloride (MgCl_2_), sodium phosphate, sodium chloride, human insulin, hydrocortisone 21-hemisuccinate (HCC), and glutamine were purchased from Sigma–Aldrich Chemie B.V. (Zwijndrecht, Netherlands). Penicillin-streptomycin solution was purchased from Invitrogen (Breda, Netherlands). Phosphate-buffered saline (PBS), trypsin EDTA (trypsin (0.025%)/EDTA (0.01%)), Dulbecco’s modified Eagle’s Medium/Ham’s nutrient mixture F12 (DMEM/F12), Phenol Red Free DMEM/F-12, fetal calf serum (FCS), dextran-coated charcoal-treated (DCC) FCS, non-essential amino acids (NEAAs), geneticin (G-418), Williams’ E medium (WEM), Phenol Red Free WEM was purchased from Gibco (Paisley, United Kingdom). Dimethyl sulfoxide (DMSO) was purchased from Acros Organics (Geel, Belgium). Low salt buffer (LSB) consisted of 10 mM Tris (Invitrogen), 2 mM dithiothreitol (DTT) (Duchefa Biochemie bv, Haarlem, Netherlands), and 2 mM 1, 2-diaminocyclohexane triacetic acid monohydrate (CDTA) (Fluka, Munich, Germany). The flashmix consisted of 20 mM tricine (Jansen chemica, Landsmeer, Netherlands), 1.07 mM (MgCO_3_)4Mg(OH)_2_.5H_2_O (Sigma-Aldrich, 99% purity), 2.67 mM magnesium sulphate (MgSO4) (Ridel de Haën, Landsmeer, Netherlands), 0.1 mM ethylenedinitrilotetraacetic acid disodium salt dihydrate (Titriplex III; Merck, Amsterdam, Netherlands), 2 mM DTT (Duchefa Biochemie), 0.47 mM D-luciferin (Duchefa Biochemie), and 5 mM adenosine-5′ -triphosphate (ATP, Boehringer, Alkmaar, Netherlands). Acetonitrile (ACN) was purchased from Biosolve (Valkenswaard, Netherlands).

### 2.2 Methods

Performing the PBK modelling-facilitated QIVIVE of FLU without and with the contribution of HF bioactivity (–HF and +HF, respectively), the following steps were defined:

1. Determination of *in vitro* concentration-response data of FLU and HF in the AR-CALUX assay.
2. PBK model development describing FLU and HF kinetics in humans.
3. Sensitivity analysis and PBK model validation with population simulation.
4. PBK modelling-facilitated QIVIVE translating the *in vitro* concentration-response data to *in vivo* dose-response data, -HF and +HF.
5. BMD analysis of the predicted dose-response data and comparison to relevant *in vivo* doses.

#### 2.2.1 Determination of *in vitro* Concentration-Response Data of FLU and HF in the AR-CALUX Assay

##### 2.2.1.1 Cell Culture

Cells from the stably transfected human osteosarcoma (U2OS) cell line expressing the human AR (BioDetection Systems (BDS), Amsterdam, Netherlands) were maintained in DMEM/F-12 supplemented with 10% FCS, 1% NEAAs, 10 units/mL penicillin, 10 µg/mL streptomycin, and 0.2 mg/mL G-418 in an incubator (37°C, 5% CO_2_, 100% humidity). The cells were routinely subcultured when reaching 85–95% confluency (*i.e.,* every 3–4 days) using trypsin-EDTA.

##### 2.2.1.2 AR-CALUX Assay

The AR-CALUX assay used to obtain the concentration-response curves of FLU and HF was performed as described previously (Sonneveld et al., 2005; van der Burg et al., 2010). Briefly, the AR-CALUX U2OS cells were plated in white, clear-bottomed 96-well plates at a density of 1*10^5^ cells/mL in a volume of 100 μL/well assay medium consisting of Phenol Red Free DMEM/F-12 supplemented with 5% DCC-FCS, 1% NEAAs, 10 units/mL penicillin, and 10 μg/mL streptomycin. The outer wells were left empty to be loaded with 200 µL PBS to prevent evaporation of the assay medium. The cells were plated for 24 h in an incubator (37°C, 5% CO_2_, 100% humidity) after which 100 µL of the assay medium was refreshed and the cells were placed again for 24 h in an incubator (37°C, 5% CO_2_, 100% humidity). Next, the assay medium was aspirated and the cells in each well were exposed for 24 h in an incubator (37°C, 5% CO_2_, 100% humidity) to 100 µL assay medium containing the assigned concentration of the corresponding compound, the exposure medium. A concentration range of DHT (0.01–100 nM) (added from 1,000 times concentrated stock solutions in DMSO, prepared in 2 mL exposure medium), the vehicle control (0.1% DMSO) and the cytotoxicity control (10 µM TBT) were tested in triplicates in the agonism assay. A concentration range of FLU (0.03–300 µM) or HF (0.001–30 M) (added from 2000 times concentrated stock solutions in DMSO, prepared in 2 mL exposure medium), the vehicle control (0.1% DMSO) and the cytotoxicity control (10 µM TBT) were all tested in triplicates in the antagonism assay. In the antagonism assay, the assay medium was supplemented with the EC_50_ (1 nM) of the agonist DHT (added from a 2000 times concentrated stock solution in DMSO, prepared in the 2 mL exposure medium). After the exposure medium was aspirated, the cells were washed with 100 µL PBS in MilliQ water (1:1) and lysed with 30 μL LSB. After a 30 min arrest on ice, plates were stored overnight in −80°C. Luminescence was measured using the GloMax 96 Microplate luminometer (Promega Benelux, Leiden, Netherlands) wherein 100 μL flash mix containing ATP and luciferin was automatically added to each well. Cytotoxicity was measured using cytotox CALUX cells (U2OS cell line expressing a constitutive active luciferase reporter gene [BDS, Amsterdam, Netherlands (Van der Linden et al., 2014)], following the same protocol. The data presented are from three independent studies executed in technical triplicates.

##### 2.2.1.3 Data Analysis

Antagonism was defined as a >20% decrease in the relative induction of the DHT induced response at a non-cytotoxic concentration of FLU or HF in the AR-CALUX cells. The test concentrations tested in the cytotox CALUX cells were similar to those tested in the AR-CALUX assay and considered as cytotoxic when the relative induction of the test condition decreased more than 15% compared to the solvent control set at 100%. For these samples the observed reduction in luminescence was considered not to be due to antagonism and excluded from the analysis. The IC_50_ values of FLU and HF were modelled with a nonlinear regression of log (inhibitor) *vs.* response (four parameters) model using GraphPad Prism 5 (GraphPad, San Diego, United States). A statistical comparison was made between the concentration-response curves of FLU and HF to check whether they are parallel. This was achieved with the option “Do the best fit values of selected parameters differ between data sets” of the nonlinear regression of log (inhibitor) *vs.* response (four parameters) model of GraphPad Prism 5.

#### 2.2.2 PBK Model Development Describing FLU and HF Kinetics in Humans

The PBK model describing FLU and HF kinetics upon FLU exposure in humans was developed using the commercially available software GastroPlus™ version 9.8 (Simulation Plus Inc., Lancaster, CA, United States). The built-in Population Estimates for Age-Related (PEAR) Physiology™ module was used to parameterize for different human physiologies for model development and validation based on available human *in vivo* pharmacokinetic data reported from literature (Radwanski et al., 1989; Doser et al., 1997) to constantly match the target population. In GastroPlus, the options are to parameterize for a population of Americans, Japanese, or Chinese. To resemble a Caucasian population used in Radwanski et al. (1989) and Doser et al. (1997), the PBK model was parameterized for an American population. The chemical-specific parameters were collected from literature, PubChem databases (Kim et al., 2016), or predicted from chemical structure with the built-in ADMET Predictor™ version 9.6 (Simulation Plus Inc., Lancaster, CA) (Table 1).

**TABLE 1:** Input parameters of the PBK model describing FLU and HF kinetics in humans. MW = molecular weight. LogP = partition coefficient. pKa = dissociation constant. P_eff_ = effective permeability. F_ub *in vivo*_ = fraction unbound *in vivo*. R_b2p_ = blood: plasma ratio.

Parameters	FLU	HF
MW (g/mol)	276.22^a^	292.21^a^
LogP	3.35^a^	2.70^a^
Solubility at 25°C (mg/mL)	5.7*10^−3^ ^b^	0.16^c^
pKa	Acid 10.54^b^	Acid 0.84^b^
	Base 0.83^b^	
P_eff_ (x 10^−4^ cm/s)	5.25^d^	
F_ub_ _*in vivo*_	0.20^b^	0.32^b^
R_b2p_	0.83^b^	0.84^b^

a Kim et al. (2016).

b ADMET predictor™.

c Wishart et al. (2007).

d Zuo et al. (2000).

The effective permeability (p_eff_) of FLU was simulated from the Caco-2 value, derived from the *in vitro* colorectal adenocarcinoma cell intestinal permeability assay (van Breemen and Li, 2005), reported by Zuo et al. (2000) using the built-in conversion equation based on the Absorption Systems Caco-2 calibration (ABSCa). The distribution of FLU and HF into tissues was assumed to be perfusion limited and the tissue: plasma partition coefficients (Kps) were calculated with the Lucakova method (GastroPlus; Rodgers et al., 2005, Rodgers and Rowland, 2006).

##### 2.2.2.1 *In Vitro* Incubations of FLU and HF to Derive Kinetic Parameters

###### 2.2.2.1.1 HLM Incubations

To obtain the Michaelis-Menten parameters for the hepatic hydroxylation of FLU to HF, FLU was incubated with human liver microsomes (HLM), pooled from 50 donors, male and female (M0317, Sigma–Aldrich Chemie B.V. Zwijndrecht, Netherlands) adapting the method described by Kang et al. (2008). Prior to the kinetic study, the incubation time and HLM concentration were optimized (data not shown) to determine the conditions where the metabolite formation was linear with time and the amount of HLM. FLU (1–50 µM final concentration added from 100 times concentrated stock solutions in DMSO) was incubated for 15 min in a water bath (37°C) in a reaction mixture consisting of 0.1 M potassium phosphate (pH 7.4), 0.8 mg/mL HLM, 1 mM NAPDH, and 5 mM MgCl_2_ in a final volume of 200 µL. Reaction mixtures wherein the volume of NADPH was replaced by an equal volume of potassium phosphate (pH 7.4) served as blanks. Prior to adding the substrate to the reaction mixtures, the mixtures were pre-incubated for 1 min in a water bath (37°C). Likewise, 1 µM FLU was incubated over time (0–30 min) in the same reaction mixtures to obtain the CL_int_ of FLU. The reactions were terminated by addition of 100 µL cold acetonitrile (ACN) followed by a 30 min arrest on ice. After centrifugation (4°C) for 10 min at 15,000 × *g* (CT 15RE, Hitachi Koki Co., Ltd.), 100 µL supernatant was collected for LC-MS/MS analysis for HF or FLU quantification, respectively. The data presented are from three independent studies executed in technical duplicates.

###### 2.2.2.1.2 HepaRG Cell Culture

To CL_int_ of HF was obtained using the hepatoma HepaRG cell line (undifferentiated HepaRG cells were purchased from Biopredic International, HPR101, p12 Rennes, France), since no clearance was observed in HLM or human S9 incubations (data not shown). In light of the scope of this work, the incubations were performed with HepaRG cells differentiated *in vitro* to hepatocyte- and cholangiocyte-like cells (1:1) (Gripon et al., 2002; Aninat et al., 2006). To this end, cryopreserved undifferentiated HepaRG cells were thawed and grown in T75 flasks in culture medium consisting of WEM supplemented with 10% FCS, 100 units/mL penicillin, 100 µg/ml streptomycin, 2 mM glutamine, 50 μM HCC, and 5 µg/ml human insulin for approximate 2 weeks and placed in an incubator (37°C, 5% CO_2_, 100% humidity). The culture medium was refreshed every 2–3 days until 80–90% confluency was reached. Then, the cells were plated at a density of 2*10^5^ cells/well in 6 well plates in a volume of 2 mL in culture medium and placed in an incubator (37°C, 5% CO_2_, 100% humidity). The culture medium was refreshed every 2–3 days until 80–90% confluency was reached before initiating the differentiation of the cells. At day 1 of the differentiation, the culture medium was supplemented with 1.7% DMSO. After two days, the culture medium was supplemented with 2% DMSO (differentiation medium) which was refreshed every 2–3 days until day 14 at which HepaRG cells are known to be fully differentiated (Gripon et al., 2002; Aninat et al., 2006).

###### 2.2.2.1.3 HepaRG Cell Incubations

The differentiated HepaRGs were washed 2 times with assay medium consisting of Phenol Red Free WEM supplemented with 100 units/mL penicillin, 100 µg/ml streptomycin, 2 mM glutamine, 50 μM HCC, and 5 µg/mL human insulin. Next, HepaRG cells were exposed to 2 mL assay medium consisting of 0.1 µM HF (final concentration added from a 1,000 times concentrated stock solution in DMSO) or the vehicle control (0.1% DMSO) in triplicate and incubated for 0, 2, 4, 6, and 24 h. After each timepoint, 100 µL supernatant was transferred to vials for LC-MS/MS analysis. A similar experiment was conducted in sync using cell free plates to serve as blanks. After the 24 h timepoint, the cells of each well were washed 2 times with 1 ml PBS and once with 0.5 mL trypsin-EDTA. After 2–3 min, the cells were resuspended with 2 mL assay medium and collected in Eppendorf tubes for cell counting using a Cellometer^®^ (Nexcelom Bioscience, Lawrence, MA, United States). The data presented are from two independent studies.

###### 2.2.2.1.4 Quantification of FLU and HF Using LC-MS/MS

The detection and quantification of FLU and HF in the supernatant following the incubations were performed using a Shimadzu LCMS-8045 mass spectrometer (Kyoto, Japan), operating under negative electrospray ionization (ESI) conditions. Chromatographic separation was performed on a Kinetic^®^ 1.7 µm C18 100 Å column (50 × 2.1 mm) (Phenomenex, Torrance, CA, United States). The column and autosampler temperature were set at 40°C and 5°C, respectively. The injection volume was 1 µL at a flow rate of 0.3 mL/min. The mobile phase A consisted of MilliQ water with 0.1% (v/v) formic acid. Mobile phase B was ACN with 0.1% (v/v) formic acid. The following gradient was used: 0–7 min linear increase from 0% B to 100% B, 7–8 min 100% B, 8–9 min back to initial conditions of 0% B. Subsequently, the column was re-equilibrated for 4 min at 0% B before the next injection. The acquisition parameters of FLU and HF are summarized in Supplementary Material S1.

###### 2.2.2.1.5 Calculation of Kinetic Parameters of FLU and HF

The Michaelis-Menten equation (Eq. 1) was used to calculate the V_max_ and K_m_ of the hydroxylation of FLU to HF by HLM.

$$\text{v}=\frac{{\text{V}}_{\text{max}}\text{∗}\left[\text{S}\right]}{\left(K_{m}+\left[S\right]\right)}$$ (1)

In this equation v represents the reaction rate expressed in nmol/min/mg microsomal protein, V_max_ the apparent maximum rate in nmol/min/mg microsomal protein, S the substrate concentration in µM, and K_m_ the Michaelis-Menten constant in µM. The calculation was executed with GraphPad Prism 5 (GraphPad, San Diego, United States). To determine the CL_int_ of FLU, a depletion curve of the measured concentrations over time following the incubation with HLM was constructed by plotting the ln(C_compound_/C_blank_) versus time. The elimination rate constant k (min^−1^) is obtained from the slope of the linear part of this depletion curve. C_compound_ and C_blank_ are the remaining concentration of the compounds after the incubation in the incubation samples or the corresponding blanks, respectively. Next the CL_int_ value of FLU (expressed in μL/min/mg microsomal protein) was calculated following Eq. 2.

$${\text{CL}}_{\text{int}}=\text{k∗}\frac{\text{V}}{\text{P}\left(\text{HLM}\right)}$$ (2)

In this formula k represents the elimination rate constant (min^−1^), V presents the incubation volume (µL) and P (HLM) the amount of microsomes (mg microsomal protein) in the incubation mixture. The V_max_ and CL_int_ following HLM incubations with FLU were scaled to whole human liver assuming an HLM protein concentration of 34 mg/g liver and a liver weight of 1.58 kg (females) or 1.84 kg (males) (GastroPlus suggested default values). To determine the CL_int_ of HF a depletion curve was constructed of the measured concentrations over time following the HepaRG incubations. The CL_int_ of HF (expressed in µL/min/million cells) was calculated following Eq. 3.

$${\text{CL}}_{\text{int}}=\text{k∗}\frac{\text{V}}{\text{P}\left(\text{cell}\right)}$$ (3)

In this equation k represents the elimination rate constant (min^−1^), V presents the incubation volume (µL) and P (cell) represents the cell amount per well expressed per million liver cells. The CL_int_ was scaled to whole human liver based on hepatocyte scaling factors (Punt et al., 2019) embodying 120 million hepatocytes/g liver and a liver weight of 1.58 kg (females) or 1.84 kg (males). It was assumed that the scaling factor expressed per million hepatocytes would be valid to translate the CL_int_ for the HepaRG liver cells to the whole liver, an assumption supported by the fact that the metabolic capacity of HepaRGs has been frequently reported to resemble that of human primary hepatocytes (Gripon et al., 2002; Zanelli et al., 2011; Punt et al., 2019).

The PBK model was parameterized for a fasted 30 year old female with a body weight of 75.57 kg to consistently match *in vivo* pharmacokinetic data reported from females by Doser et al. (1997). Simulations were carried out and the V_max_ of FLU hydroxylation to HF was further optimized by visual examination until the prediction of the time-dependent plasma concentrations of FLU and HF consistently matched the *in vivo* pharmacokinetic data (Doser et al., 1997) to confirm the model development.

#### 2.2.3 Sensitivity Analysis and PBK Model Validation With Population Simulation

##### 2.2.3.1 Sensitivity Analysis

A sensitivity analysis was performed to indicate which parameters are most influential on the prediction of the maximum plasma concentration (C_max_) and area under the concentration time curve (AUC) of FLU and HF upon an oral dose regimen of 250 mg FLU at the first day and 250 mg three times a day through day 2–8, later denoted as the repeated dose model (Radwanski et al., 1989). The PBK model was parameterized for a 30 year old American male with a body weight of 70 kg, to estimate a standard human (Brown et al., 1997), and the sensitivity analysis was executed with the built-in parameter sensitivity analysis (PSA) mode of GastroPlus. The sensitivity coefficients (SCs) for the C_max_ and AUC of FLU and HF were calculated as the % change in model outcome divided by the % change in parameter value (Eq. 4).

$$\text{SC}=\frac{\text{\% change in model outcome}}{\text{\% change in parameter value}}$$ (4)

The % change in parameter value was set at 5% for one parameter at a time (Zhang Q. et al., 2018; Moxon et al., 2020). Parameters with a SC > 0.1 or < −0.1 were considered to be influential on the prediction of the C_max_ and AUC of FLU and HF (Zhang M. et al., 2018).

##### 2.2.3.2 PBK Model Validation With Population Simulation

Next, the developed PBK model describing FLU and HF kinetics in humans upon FLU exposure was parameterized for a 66 year old male with a body weight of 89 kg for validation of the predictions by the repeated dose model, an oral dose regimen of 250 mg FLU at the first day and 250 mg three times a day through day 2–8, against reported data following repeated exposure (Radwanski et al., 1989). Population simulation of the repeated dose model in humans was carried out using the GastroPlus built-in population simulator, based on the Monte Carlo method, to obtain the distribution in the predicted time-dependent plasma concentrations of the FLU and HF over a healthy American population. Default distributions of the Population Estimates for Age Related Physiology (PEAR) were used for an American population of 100 healthy American (with 50: 50 ratio of male: female) of 20–80 years old with a body weight of 50–110 kg. The number of iterations was set at 300 and simulation time at 288 h to reach the C_max_ values. The PBK model is defined valid when the predicted FLU and HF kinetics in humans are within the acceptance criteria predicting the C_max_ values within a 2-fold difference of the corresponding literature reported C_max_ values (Jones et al., 2015).

#### 2.2.4 PBK Modelling-Facilitated QIVIVE Translating the *In Vitro* Concentration-Response Data to *In Vivo* Dose-Response Data, − and +HF

PBK modelling-facilitated QIVIVE was performed to translate the *in vitro* AR-CALUX derived concentration-response curve of FLU to the corresponding *in vivo* dose-response curves, either without or with taking the effect of HF into account (−HF and +HF). The PBK model was parameterized for a 30 year old American male with a body weight of 70 kg to estimate a standard human (Brown et al., 1997). Simulations were carried out with the repeated dose model with a simulation time of 288 h in order to reach steady state of the C_max_. In the QIVIVE, it is assumed that the free *in vitro* effect concentrations are equal to the free *in vivo* C_max_.

##### 2.2.4.1 QIVIVE −HF

Performing the QIVIVE –HF, the nominal concentrations of FLU from the *in vitro* AR-CALUX assay were corrected for *in vitro* protein binding to obtain the free *in vitro* concentrations, following Eq. 5.

$$\text{free }in\;vitro\text{ concentration FLU}=\text{nominal }in\;vitro{\text{ concentration}\text{ FLU ∗ f}}_{\text{ub}\;in\;vitro,\;\text{FLU}}$$ (5)

The nominal *in vitro* concentrations of FLU were derived from the AR-CALUX assay and the f_ub_ _*in vitro*, FLU_ represents the fraction unbound in the medium used in the AR-CALUX assay amounting to 0.50 for FLU (Van Tongeren et al., 2021). Next, the free *in vitro* concentrations of FLU were assumed equal to the free C_max_ values of FLU at steady state. Using the developed PBK model, the FLU doses were simulated that are required to reach the corresponding free C_max_ values at steady state, generating the dose-response curve for the anti-androgenic activity of FLU -HF.

##### 2.2.4.2 QIVIVE +HF

Performing the QIVIVE of the *in vitro* AR-CALUX derived concentration-response curve to generate a dose-response curve for the anti-androgenic effect of FLU taking the activity of HF into account, a toxic equivalency factor (TEF) approach (Zhao et al., 2021) was included in the PBK model to predict the combined free C_max_ values of FLU and HF expressed in FLU equivalents (Eq. 6).

$${\text{Combined free C}}_{\text{max}}\text{ of FLU and HF expressed in FLU equivalents}={\text{C}}_{\text{max},\;\text{FLU}}*{\text{f}}_{\text{ub}\;in\;vivo,\;\text{FLU}}*{\text{TEF}}_{\text{FLU}}+{\text{C}}_{\text{max},\;\text{HF}}*{\text{f}}_{\text{ub}in\;vivo,\;\text{HF}}*{\text{TEF}}_{\text{HF}}$$ (6)

The C_max, FLU_ and C_max, HF_ are the maximum plasma concentration of FLU and HF, respectively. The f_ub_ _*in vivo*, FLU_ and the f_ub_ _*in vivo*, HF_ are the fraction unbound *in vivo* of FLU and HF (Table 1). The TEF_FLU_ and TEF_HF_ correspond to the toxic equivalency factor of FLU and HF, respectively. The TEF_FLU_ was equalized to 1.0 whereas TEF_HF_ was calculated following Eq. 7.

$${\text{TEF}}_{\text{HF}}=\frac{{\text{IC}}_{50}\text{FLU}}{{\text{IC}}_{50}\text{HF}}$$ (7)

To use this TEF approach, 3 criteria need to be met (Zhao et al., 2021). First, FLU and HF act *via* the same mode of action. Second, the concentration-response curves in the AR-CALUX assay of FLU and HF are parallel. Third, the toxicity of FLU and HF in the AR-CALUX assay is additive. If the data are compliant to these criteria, QIVIVE +HF is performed. The free *in vitro* concentrations of FLU obtained from the *in vitro* AR-CALUX assay were then set equal to the combined free C_max_ of FLU and HF expressed in FLU equivalents in the PBK model. Next, the FLU doses that are required to obtain the corresponding combined free C_max_ of FLU and HF expressed in FLU equivalents were simulated using the PBK model. This generates the dose-response curve of the anti-androgenic activity of FLU +HF.

#### 2.2.5 BMD Analysis of the Predicted Dose-Response Data and Comparison to Relevant *In Vivo* Doses

BMD analysis was performed for the predicted dose-responses of FLU − and +HF to define the BMDL_05,_ and the upper bound of the 95% confidence interval of the benchmark dose at a 5% extra response compared to the background (BMDU_05_) values using the BMDS3.2.1 software (US EPA). When the BMDU_05_: BMDL_05_ ratio (precision factor) was below 3 and the *p*-value > 0.05, support for a dose-response was indicated and the BMDL_05_ value was accepted (US Environmental Protection Agency, 2012; European Food Safety Authority et al., 2017). The BMDL_05_ values were then compared to the therapeutic dose of 250 mg FLU 3 times per day for the treatment of prostate cancer (Schellhammer et al., 1997) and 125 mg FLU per day for the treatment of hirsutism (Calaf et al., 2017). Furthermore, a comparison was made with PoDs defined for FLU exposure. To this end, a literature search was conducted to collect available. So include a PODs to FLU exposure from animal studies. Then it was checked whether these studies comply with the most up to date evaluation and assessment criteria of the current testing guidelines and whether the same conclusion in terms of the reference values could be made. Only the no observed adverse effect level (NOAEL) values obtained from the studies that met these criteria (Zacharia, 2017) were used for comparison, following the OECD protocol 407 for a 28 days toxicity study in rats incorporating the Hershberger bioassay (OECD, 2008), the OECD protocol 441 for the Hershberger bioassay in rats (OECD, 2009), or the OECD protocol 421 for the Reproduction/Developmental Toxicity Screening Test (OECD, 2016).

## 3 Results

### 3.1 Determination of *in vitro* Concentration-Response Data of FLU and HF in the AR-CALUX Assay

**FIGURE 2:**
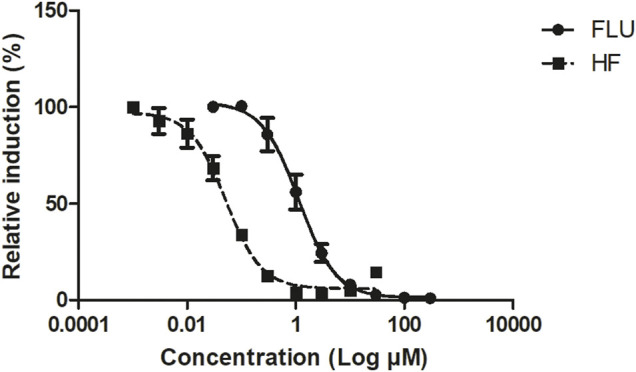
The concentration-dependent antagonistic activity of FLU (solid line and circles) and HF (dashed line and squares), on the DHT-mediated luciferase induction in the U2OS AR-CALUX reporter gene assay. The symbols present the mean ± SD values of 3 independent studies.

The *in vitro* concentration-response curves for the anti-androgenic activity of FLU and HF in the AR-CALUX assay are depicted in Figure 2. The nominal IC_50_ values of FLU and HF equalled 1.14 and 0.05 µM, respectively. The statistical comparison between the concentration-response curves of FLU and HF confirmed that they run parallel with a hillslope of −1.247 and −1.354, respectively (*p* value = 0.6985).

### 3.2 PBK Model Development Describing FLU and HF Kinetics in Humans

To enable PBK modelling-facilitated QIVIVE of the anti-androgenic response of FLU, - and +HF, a PBK model was developed describing FLU and HF kinetics in humans. Parameters describing hepatic metabolism of FLU and HF were determined *in vitro*.

#### 3.2.1 *In vitro* Incubations of FLU and HF to Derive Kinetic Parameters

**FIGURE 3:**
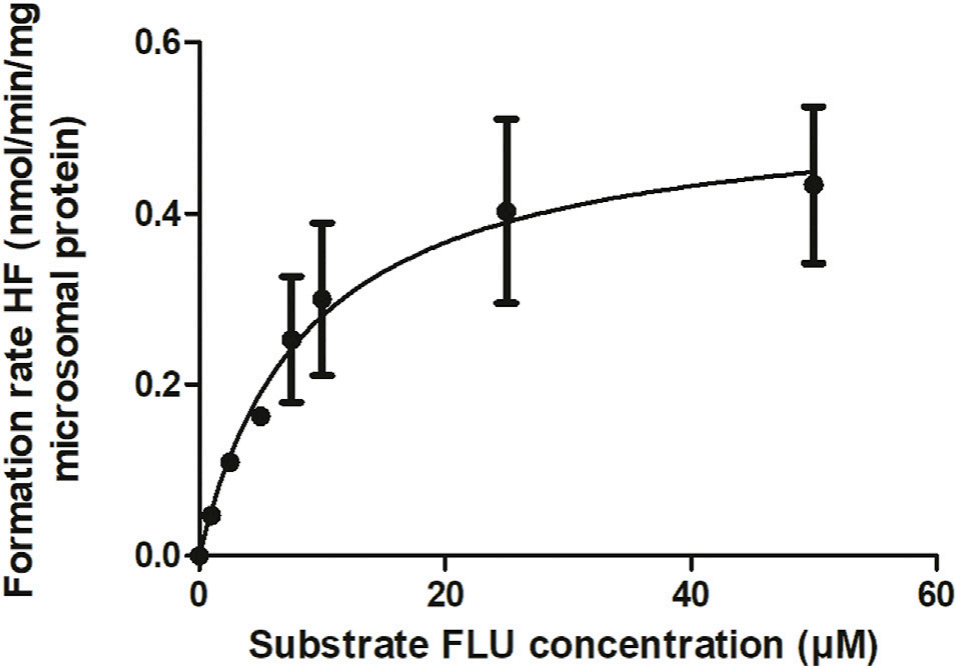
CYP-mediated formation rate of HF following HLM incubations with FLU. The symbols present the mean ± SEM values of 3 independent studies.

The kinetic parameters for the hepatic hydroxylation of FLU to HF were obtained by incubation of FLU with pooled HLM. Figure 3 shows the Michaelis-Menten kinetics of FLU conversion to HF. The corresponding V_max_ and K_m_ values and the HLM incubation derived CL_int_ value of FLU are summarized in Table 2. The V_max_ was further optimized by visual examination until the prediction of the time-dependent plasma concentrations of FLU and HF consistently matched the *in vivo* pharmacokinetic data (Doser et al., 1997) (Figure 5A).

**TABLE 2:** Kinetic parameters of hepatic metabolism of FLU and HF.

Kinetic parameter	Value *in vitro*
V_max_ FLU to HF	0.53 ± 0.08 nmol/min/mg protein
Optimized V_max_ FLU to HF^a^	0.27 nmol/min/mg protein
K_m_ FLU to HF	8.85 ± 3.64 µM
CL_int_ FLU	116.63 ± 15.61 µL/min/mg protein
CL_int_ HF	10.18 ± 0.50 µL/min/million cells

a Optimized value by visual examination until the prediction of the time-dependent plasma concentrations of FLU and HF consistently matched the *in vivo* pharmacokinetic data (Doser et al., 1997) (Figure 5A).

The CL_int_ value of HF was obtained following incubations with HepaRGs (Table 2). The cell count after 24 h of HF incubation with HepaRGs revealed 0.61 million cells/incubation and this value was used to calculate the CL_int_ of HF. All kinetic values were scaled to whole human liver in the PBK model as described in the Materials and methods section.

### 3.3 Sensitivity Analysis and PBK Model Validation With Population Simulation

#### 3.3.1 Sensitivity Analysis

**FIGURE 4:**
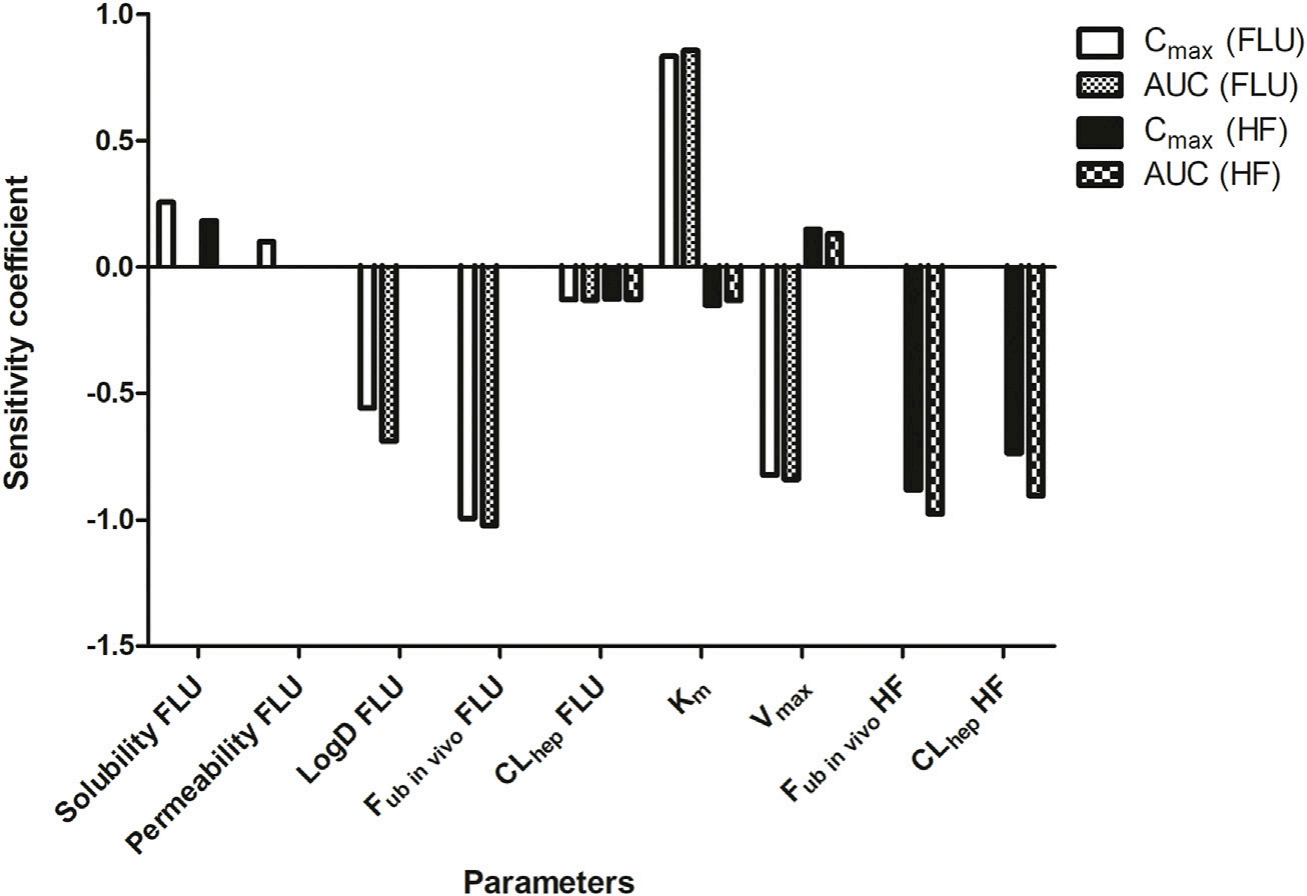
Sensitivity analysis of the parameters of the PBK model describing FLU and HF kinetics in humans by the repeated dose model. Only parameters with a SC > 0.1 or < −0.1 for predicting the C_max_ and AUC of FLU and HF are presented. Permeability = intestinal permeability. LogD = distribution coefficient. F_ub_ _*in vivo*_ = fraction unbound *in vivo*. CL_hep_ = hepatic clearance. V_max_ = V_max_ of FLU conversion to HF. K_m_ = K_m_ of FLU conversion to HF.

The PBK model was parameterized for a 30 year old American male with a body weight of 70 kg to estimate a standard human (Brown et al., 1997) and the sensitivity analysis was conducted on the repeated dose model for evaluation. Figure 4 depicts the SCs of parameters as identified being most influential (SC > 0.1 or < −0.1) on the model outcomes for C_max_ and the AUC of FLU and HF.

The PBK model prediction of the C_max_ of FLU is sensitive to the solubility, permeability, LogD, f_ub_ _*in vivo*,_ and CL_hep_ of FLU, the V_max_ and K_m,_ and the f_ub_ _*in vivo*_ and CL_hep_ of HF. The prediction of the AUC of FLU is sensitive to the LogD, f_ub_ _*in vivo*,_ and CL_hep_ of FLU, and the V_max_ and K_m._ Influential parameters on the prediction of the C_max_ and AUC of HF are the CL_hep_ of FLU, the V_max_ and K_m,_ and the f_ub_ _*in vivo*_ and CL_hep_ of HF.

#### 3.3.2 PBK Model Validation With Population Simulation

**FIGURE 5:**
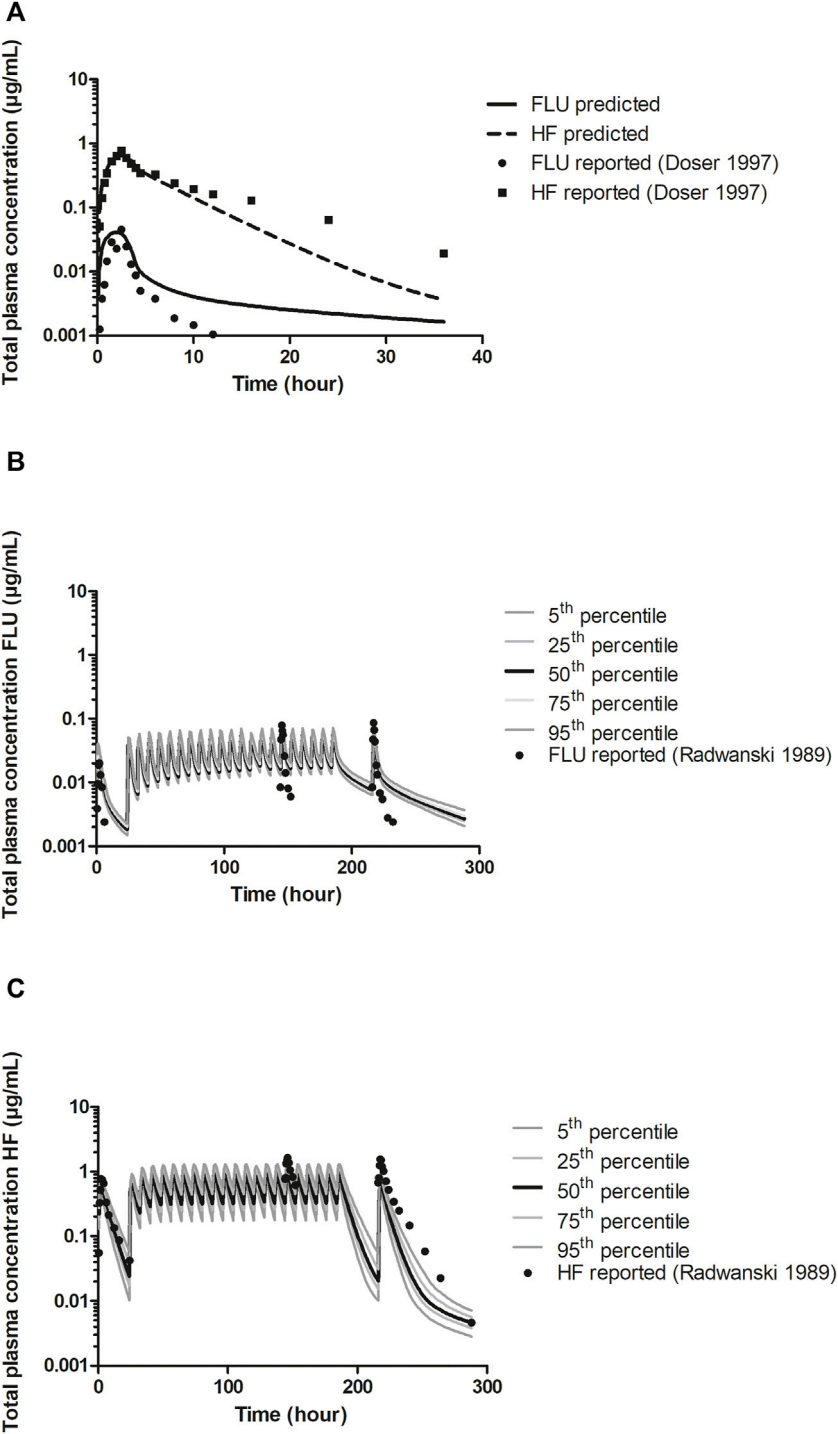
**A)** PBK model predicted (line and dashed line) and reported (circles and squares) time-dependent total plasma concentrations of FLU and HF following a single oral dose of 250 mg FLU (experimental data from Doser et al., 1997) in humans for model development. Prediction was obtained after optimization of the V_max_ against reported data (Doser et al., 1997). **(B)** and **(C)**. PBK model predicted and reported (circles) time-dependent total plasma concentrations of FLU and HF, respectively, following an oral dose regimen of 250 mg FLU at the first day and 250 mg three times a day through day 2–8 (repeated dose model) (experimental data from Radwanski et al., 1989) for model validation, including the distribution of the predictions among an American healthy population. The 5th and 95th percentiles and the 25th and 75th percentiles of the predictions are presented as dark grey and light grey lines, respectively, the 50th percentile presented by the black lines.

To further evaluate the developed PBK model describing FLU and HF kinetics in humans with the optimized V_max_ value of FLU conversion to HF, mode predictions were compared with reported human *in vivo* pharmacokinetic data (Radwanski et al., 1989; Doser et al., 1997). Figure 5A shows the predicted and literature reported time-dependent total plasma concentrations of FLU and HF following a single oral dose of 250 mg FLU. Figures 5B,C show the predicted and literature reported time-dependent total plasma concentrations following the repeated dose model, including the distribution of the predictions over a healthy American population. Comparison indicates that the PBK model predicts the time-dependent total plasma concentrations of FLU and HF within the acceptance criteria, i.e., predicting the C_max_ values within a 2-fold difference of the corresponding literature reported C_max_ values (Jones et al., 2015). Furthermore, the distribution of the predicted plasma concentrations of FLU and HF following the repeated dose model in a healthy American population was quantified by dividing the 95th percentile by the geometric mean amounting to 1.22 µg/mL and 1.37 µg/mL and of FLU and HF respectively. Additionally, the coefficient of variation (CV) which compares the standard deviation to the mean of predicted time-dependent total plasma concentrations was calculated amounting to 13% and 23% for FLU and HF, respectively. This indicates there is a somewhat wider distribution of the HF plasma concentrations in the PBK model predictions than of the FLU concentrations.

### 3.4 PBK Modelling-Facilitated QIVIVE Translating the *in vitro* Concentration-Response Data to *in vivo* Dose-Response Data, − and +HF

This work is compliant to the three criteria set since, firstly, FLU and HF both inhibit the AR (Figure 2). Secondly, the concentration-response curves of FLU and HF in the AR-CALUX are parallel. Thirdly, the toxicity of FLU and HF in the AR-CALUX are additive (Supplementary Material S2).

#### 3.4.1 QIVIVE–and +HF

**FIGURE 6:**
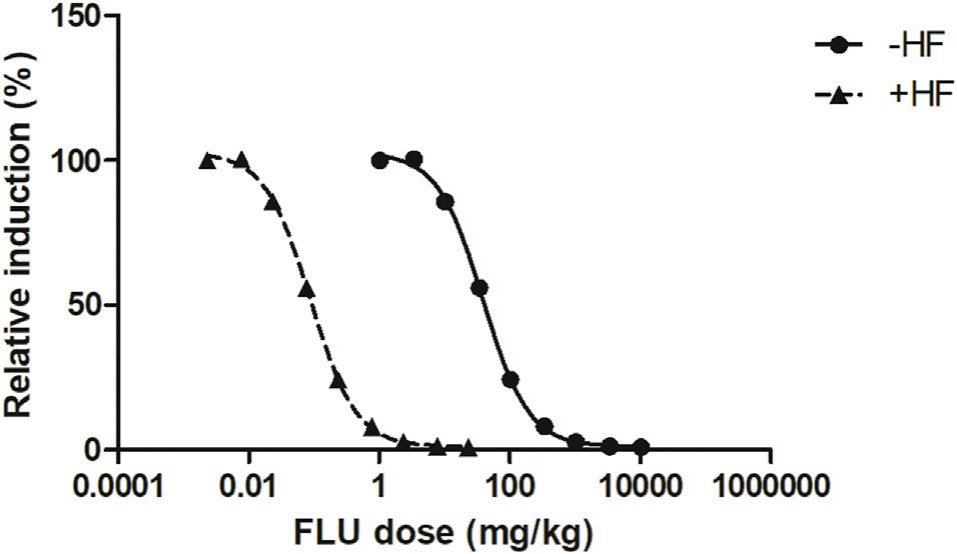
The PBK modelling-facilitated QIVIVE predicted *in vivo* dose-dependent anti-androgenic effects following FLU exposure −HF (solid line and triangles) and +HF (dashed line and squares) in humans.

The free *in vitro* concentrations of FLU were obtained by correcting for protein binding. These were set equal to the free C_max_ of FLU or the combined free C_max_ of FLU and HF expressed in FLU equivalents, the TEF_FLU_ being set at 1 and the TEF_HF_ calculated as 23 (Eq. 7). Using the developed PBK model, the corresponding FLU doses to reach those C_max_ values were predicted. Figure 6 shows the predicted *in vivo* dose-response curve for the anti-androgenic effects following FLU exposure in humans, −HF and +HF. A clear left-shift in the predicted dose-dependent anti-androgenic effect of FLU is observed, indicating that FLU appears to be more potent once the formation and activity of HF is taken into account.

### 3.5 BMD Analysis of the Predicted Dose-Response Data and Comparison to Relevant *in vivo* Doses

**FIGURE 7:**
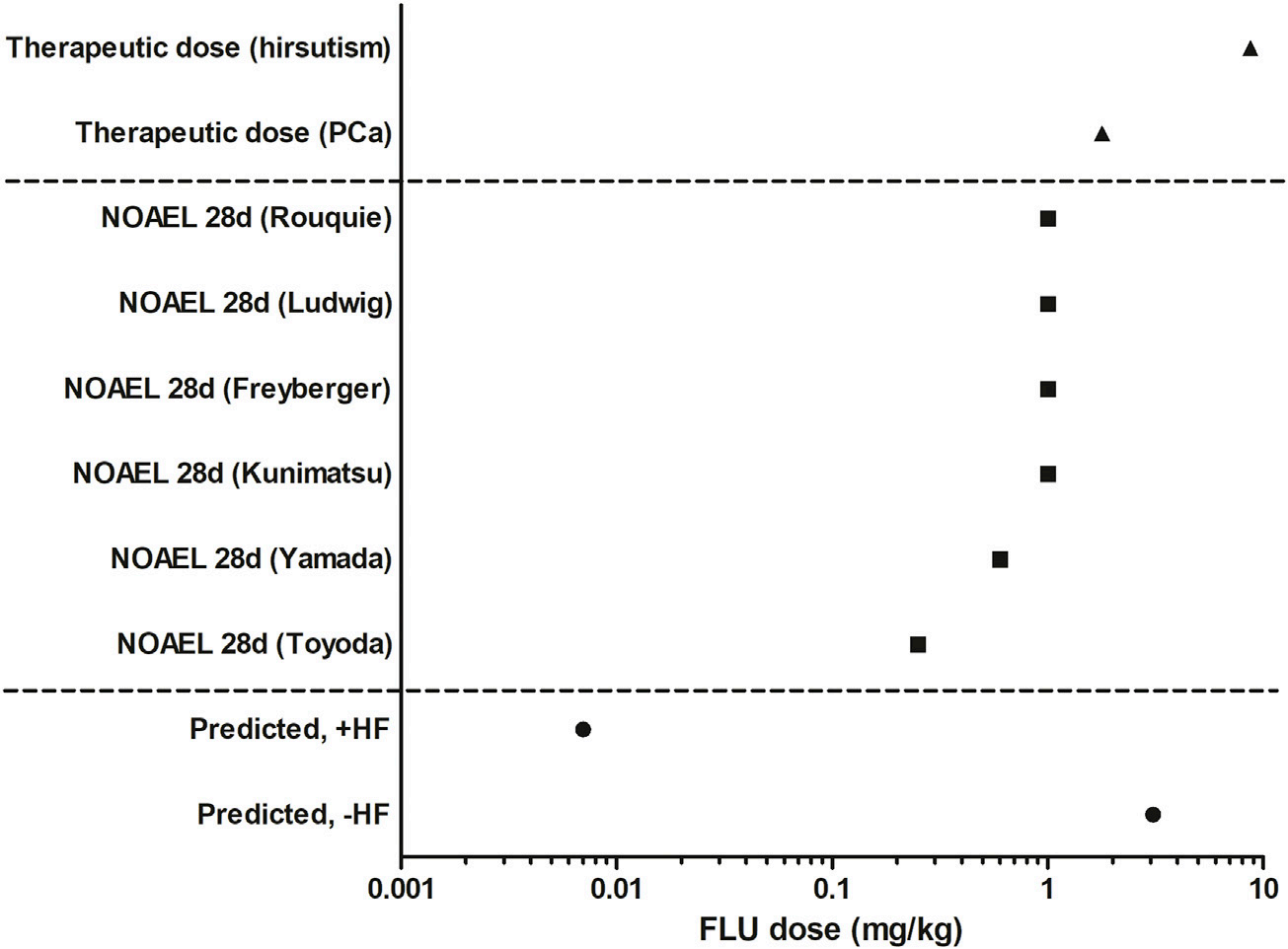
Comparison of the predicted BMDL_05_ of FLU −HF and +HF (circles), therapeutic active doses of FLU (triangles, Schellhammer et al., 1997; Calaf et al., 2017), and historical animal derived NOAELs of FLU (squares, Toyoda et al., 2000; Yamada et al., 2000; Freyberger et al., 2003; Kunimatsu et al., 2004; Rouquié et al., 2009; Ludwig et al., 2011; Zacharia, 2017).

To evaluate the predicted dose-dependent anti-androgenic effects of FLU, − and +HF, BMD analysis was performed (Supplementary Material S3). The predicted BMDL_05_ of the anti-androgenic effects of FLU −HF and +HF amounted to 3.08 mg/kg and 0.007 mg/kg, respectively. This indicates that when including the activity of HF in the PBK model, QIVIVE of the *in vitro* anti-androgenic response of FLU results in a BMDL_05_ value that is 440-fold lower compared to the value obtained when the activity of HF is excluded. Such a difference can be expected given that HF was 23 times more potent in the *in vitro* AR-CALUX assay and has an approximately 20 times higher plasma peak concentrations than FLU following FLU exposure in humans due to the rapid hydroxylation of FLU to HF (Doser et al., 1997). In Figure 7, the BMDL_05_ values obtained for FLU were compared to the therapeutic dose of FLU for the treatment of prostate cancer or hirsutism and the NOAELs of FLU derived from historical 28 days repeated dose toxicity studies in rats (Figure 7) (Schellhammer et al., 1997; Toyoda et al., 2000; Yamada et al., 2000; Freyberger et al., 2003; Kunimatsu et al., 2004; Rouquié et al., 2009; Ludwig et al., 2011; Calaf et al., 2017; Zacharia, 2017).

The PBK model-facilitated QIVIVE of the *in vitro* anti-androgenicity of FLU -HF results in a BMDL_05_ comparable to the therapeutic doses of FLU, indicating that this may not be protective in humans given that at the therapeutic doses of FLU anti-androgenic effects are expected and that in reality HF will also contribute. This is corroborated by the fact that the PBK modelling-facilitated QIVIVE of the *in vitro* anti-androgenicity of FLU +HF results in a BMDL_05_ value substantially (i.e., 2 to 3 orders of magnitude) lower than the therapeutic dose levels. This BMDL_05_ value is also 35-fold lower than the lowest reported NOAEL from a historical 28 days *in vivo* study in rats (Toyoda et al., 2000). Together, this could suggest that a PoD based on this BMDL_05_ for FLU +HF would be health protective in humans for *in vivo* anti-androgenic responses, whereas a PoD based on the BMDL_05_ for FLU -HF would potentially underestimate the risk given that it is comparable to the therapeutic dose and higher than the historical animal derived NOAELs of FLU.

## 4 Discussion

In NGRA, safe levels of human chemical exposures are assured *via in vitro* and *in silico* approaches, without the use of animal testing. However, using *in vitro* bioactivity assays to quantify the chemical-dependent response might not always represent the corresponding *in vivo* response in the human body, since in the *in vitro* bioassay effects of toxicokinetics, such as biotransformation, are generally not included. In this work, we aimed to include the contribution of the bioactivity of HF in the PBK modelling-facilitated QIVIVE of the anti-androgenic activity of FLU using the *in vitro* AR-CALUX assay in order to set the PoD for safety assessment.

The parameters of the hepatic metabolism of FLU and HF in the PBK model development were determined *in vitro*. It is worth noting that large interindividual variation has been observed in protein content and metabolic activities in microsomes from human liver samples (Chiba et al., 2009; Zhang et al., 2015) plus, microsomal incubations are prone to inter-laboratory variation (Chiba et al., 2009). The HLM derived V_max_ of FLU hydroxylation to HF amounting to 0.53 ± 0.08 nmol/min/mg protein was approximately 3-fold higher than the corresponding literature reported value amounting to 0.16 ± 0.07 nmol/min/mg protein (Goda et al., 2006). The derived K_m_ of 8.85 ± 3.64 µM was in concordance with the reported values derived from supersomes expressing CYP1A2 amounting to 18 ± 7.50 µM (Rochat et al., 2001) and from purified fusion protein containing CYP1A2 amounting to 6 ± 0.50 µM (Shet et al., 1997). Based on the sensitivity analysis, the V_max_ of FLU appeared to be influential on both FLU and HF kinetics. Given these results, the V_max_ of FLU was further optimized against the *in vivo* data of Doser et al. (1997), resulting in an optimized V_max_ of 0.27 nmol/min/mg protein, a value intermediate between our value and that previously reported in the literature (Goda et al., 2006). This resulted in an adequate PBK model able to predict the time-dependent plasma concentrations of FLU and HF in human following repeated exposure to FLU (Figures 5B,C) (Radwanski et al., 1989). The PBK model developed describing FLU and HF kinetics in humans was also considered adequate to perform the QIVIVE of the *in vitro* anti-androgenic response of FLU.

Chemicals may bind to constituents in the surrounding medium which influences their availability for the biological target and the corresponding potency (Gülden et al., 2002). Therefore, the free concentration of a chemical is considered to be a more appropriate dose metric than the nominal concentration. It was assumed that proteins present in the media were of major influence on the free concentrations of FLU and HF. Therefore, the QIVIVE was based on the free concentrations of the FLU and HF in the *in vitro* medium and *in vivo* plasma which were obtained by correction for protein binding.

Ideally, for evaluation purposes, the BMDL_05_ derived from PBK modelling-facilitated QIVIVE of FLU −/+HF could be compared to non-anti-androgen active levels of FLU exposure in a healthy population. However, such data were not available so the BMDL_05_ was compared to the therapeutic active doses of FLU for treating prostate cancer or hirsutism based on its anti-androgenic effect (Schellhammer et al., 1997; Calaf et al., 2017). The BMDL_05_ from QIVIVE of FLU –HF appeared to be 440-fold higher than the BMDL_05_ obtained for FLU +HF which takes the activity of HF into account. The predicted BMDL_05_ value for FLU +HF is 35-fold lower than the lowest reported NOAEL from a historical 28 days *in vivo* study in rats (Toyoda et al., 2000), indicating it is likely to be protective of health in humans, especially after taking potential uncertainty factors (UFs), such as an UF for interindividual variation, into account. Not taking the HF contribution into account would result in a BMDL_05_ and thus a PoD that appears not to be sufficiently conservative. This highlights the importance of the contribution of HF to the *in vivo* anti-androgenic activity of FLU and of including the toxicokinetics and toxicodynamics of an active metabolite in the *in vitro* to *in vivo* extrapolation to derive PoDs.

The observation that the BMDL_05_ value resulting from QIVIVE for FLU +HF is 35-fold lower than the lowest reported animal-based PoD, the NOAEL from a historical 28 days repeat dose toxicity study in rats reported by Toyoda et al. (2000), might be due to kinetic species differences. Although CYP1A2 is the main enzyme responsible for the conversion of FLU to HF in both rat and humans (Shet et al., 1997; Chang et al., 2000), the rat liver microsomal (RLM) incubation derived *in vitro* V_max_ of FLU hydroxylation to HF amounting to 0.063 ± 0.008 nmol/min/mg protein (Chang et al., 2000) appears to be 4-fold lower than the HLM derived and optimized *in vitro* V_max_ for FLU hydroxylation to HF of 0.27 nmol/min/mg protein obtained in this work. Furthermore, the rat S9 derived *in vitro* CL_int_ of FLU of 4.6 µL/min/mg protein (Fabian et al., 2019) is over 400-fold lower than the in this work HLM derived *in vitro* CL_int_ of FLU of 116.63 ± 15.61 µL/min/mg protein. The slower metabolic rate for conversion of FLU to HF and the slower overall clearance of FLU in rats can be expected to result in a species difference in the *in vivo* toxicity following FLU exposure because it would result in potentially higher steady state plasma levels of the active HF metabolite at equal dose levels in human than in rats, resulting in anti-androgenic effects in human at potentially lower dose levels of FLU. Thus, HF levels in humans are suspected to be higher compared to rats at similar exposure levels and bioavailability. This could explain why the predicted PoD of FLU is lower than the animal derived PoD obtained from literature. Indeed, when in the human PBK model the V_max_ was exchanged for the RLM derived V_max_, the derived BMDL_05_ from the QIVIVE of FLU +HF amounted to 0.014 mg/kg. This BMDL_05_ is only 17-fold lower than the lowest reported animal-based PoD (Toyoda et al., 2000), illustrating that the differences in kinetics between rat and humans accounts for a substantial part of the difference between the predicted PoD for human and the animal derived PoD of FLU. Since the aim of NGRA is not to predict animal-based PoDs but to protect human health, the QIVIVE of FLU +HF is supportive of the NGRA strategy to assure human safety.

The observation that *in vitro* derived PoDs can be lower than animal derived PoDs was also reported in a study of Paul Friedman et al. (2020). In this study, 89% of *in vitro* derived PoDs were lower than the traditional animal derived PoDs for different compounds and endpoints. An explanation of this difference stated that an *in vitro* bioactivity assay measures disruption at a molecular level whereas the animal-based PoDs reflect disruption at tissue or organ level (Paul Friedman et al., 2020). Similarly, in our study, the *in vitro* derived PoD was based on chemical induced disturbances in AR-dependent transcriptional activity which was compared to animal derived PoDs based on chemical induced disturbances on body or organ weight. This may further explain the 35-fold difference between the *in vitro-* and animal-based PoDs. Consequently, the PoD from the *in vitro* AR-CALUX assay is more conservative when used in a risk assessment relative to animal-based PoDs, so that a decision based on the *in vitro* derived PoD can be considered health protective for humans.

Using *in vitro* derived PoDs instead of animal derived PoDs for toxicological risk assessment would necessitate a re-evaluation of the use of UFs (Kramer et al., 2021). The use of the UF for interspecies differences could be eliminated since the *in vitro* derived PoDs are based on human cell lines and human data. However, a different UF could be included to cover the uncertainties in NGRA being based on *in silico* and *in vitro* data, while an UF for interindividual differences in both kinetics and dynamics should also be considered. Contrary, Baltazar et al. (2020) reported *in vitro* derived PoDs which were at least as protective as corresponding animal-based PoDs, indicating the NGRA may not need the use of UFs. PBK modelling predicting chemical levels in different human populations including sensitive groups such as children and pregnant women could further help in the estimation of an adequate UF for these interindividual differences in kinetics when using an *in vitro* derived PoD in NGRA.

The 440-fold lower BMDL_05_ value from QIVIVE of FLU +HF as compared to the BMDL_05_ value from QIVIVE of FLU -HF reveals that HF substantially contributes to the anti-androgenic response following FLU exposure. Comparison of this 440-fold difference to the TEF_HF_ being 23 further highlights that in addition to a difference in toxicodynamics of the metabolite and the parent compound also differences in their kinetics contribute to the difference in the overall BMDL_05_ −HF and +HF. Thus, including PBK modelling in QIVIVE to also capture the contribution in toxicokinetics of the metabolite appears essential to set an adequate PoD. FLU is designed as a prodrug for HF and therefore it could be expected upfront that including HF in the PBK modelling-facilitated QIVIVE of FLU has a substantial effect. However, also for different types of chemicals, for which this information may be unknown, this approach will provide quantitative insights into the contribution of metabolites to both toxicokinetics and toxicodynamics following exposure to the parent compound.

In conclusion, the combined *in vitro* PBK modelling-facilitated QIVIVE provides a NAM to characterise the role of metabolism to the metabolite HF in the *in vivo* anti-androgenic responses of FLU. This presents a strategy to include toxicodynamics and toxicokinetics of relevant metabolites when defining *in vitro* derived PoDs in the NGRA evaluation of a parent compound.

## Data Availability

The original contributions presented in the study are included in the article/Supplementary Material, further inquiries can be directed to the corresponding author.
