# Supplementary material for: Next Generation Risk Assessment of the Anti-Androgen Flutamide Including the Contribution of Its Active Metabolite Hydroxyflutamide
Source: Front Toxicol. 2022 Jun 2;4:881235. doi: 10.3389/ftox.2022.881235 (PMC9201820; doi:10.3389/ftox.2022.881235)
Supplement: Supplementary file 1 [file DataSheet1.docx]

Supplementary Materials

# Supplementary materials

## Supplementary material S1

LC-MS/MS acquisition parameters

| Compound | Precursor ion (m/z) | Product ion (m/z) | Collison energy (V) | Retention time (min) |
| --- | --- | --- | --- | --- |
| FLU | 275.25 | 202.1 205.1 186.1 | 24 22 32 | 7.38 |
| HF | 291.25 | 205.00 175.05 155.05 | 20 31 38 | 6.87 |

## Supplementary material S2


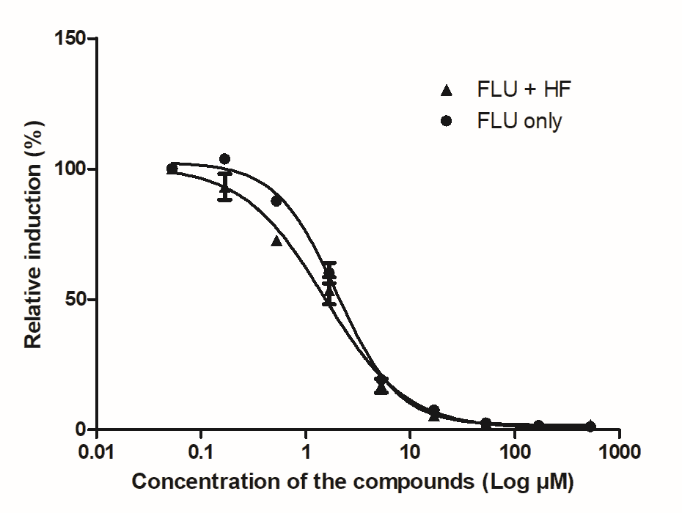


**Supplementary Figure S1.2.** The concentration-dependent antagonistic activity of FLU only (solid line and circles) and an equipotent mixture of FLU + HF (solid line and triangles) on the DHT-mediated luciferase induction in the U2OS AR-CALUX reporter gene assay. The symbols present the mean ± SD values of 3 independent studies. The activity obtained in the presence of 1 nM DHT was set at 100%. The IC_50_ values of FLU only and the equipotent mixture of FLU +HF were calculated as 1.51 and 1.98 µM, respectively.

## Supplementary material S3

The BMD analysis of the predicted anti-androgenic response of FLU – and +HF was performed using BMDS3.2.1 software (U.S. EPA). The benchmark response (BMR) was defined as a 5% extra response (BMR05). The BMC05 and its upper (BMCU05) and lower (BMCL05) 95% confidence interval were also determined. The model was accepted when the fitted model had a p-value > 0.05, a BMDU_05_: BMDL_05_ ratio (precision factor) below 3, or the lowest AIC, indicating support for a concentration-response.

### Supplementary material S3.1. BMD modelling of the predicted anti-androgenic response of FLU -HF

#### Supplementary Table S1.3.1.1. Input values of the predicted dose-response data of FLU -HF.

| Dose (mg/kg) | n | response | SD |
| --- | --- | --- | --- |
| 1.03 | 3.00 | 100.00 | 0.00 |
| 3.42 | 3.00 | 100.47 | 3.61 |
| 10.27 | 3.00 | 85.84 | 14.98 |
| 34.25 | 3.00 | 56.06 | 15.64 |
| 102.74 | 3.00 | 24.46 | 8.03 |
| 342.47 | 3.00 | 8.18 | 4.42 |
| 1027.40 | 3.00 | 2.78 | 1.37 |

**Supplementary Table S1.3.1.2.** BMD analysis of the predicted dose-response data of FLU –HF. BMD_05_, BMDL_05,_ and BMDU_05_ values were obtained using BMDS software version 3.2.1, at a BMD of 5% extra risk, BMR type Relative Deviation with normal distribution and constant variance.

| Model | BMD (mg/kg) | BMDL (mg/kg) | BMDU (mg/kg) | Test 4 P‑Value | AIC | Accepted |
| --- | --- | --- | --- | --- | --- | --- |
| [Exponential 2 (CV - normal)](file:///C:\Users\tonge015\AppData\Local\Microsoft\Windows\INetCache\Content.MSO\CABDB153.xlsx#'freq-exp2-rest-opt1'!A1) | 0.066 | 0.000 | 0.079 | <0.0001 | 251.490 | No |
| [Exponential 3 (CV - normal)](file:///C:\Users\tonge015\AppData\Local\Microsoft\Windows\INetCache\Content.MSO\CABDB153.xlsx#'freq-exp3-rest-opt1'!A1) | 0.066 | 0.000 | 0.079 | <0.0001 | 251.490 | No |
| [Exponential 4 (CV - normal)](file:///C:\Users\tonge015\AppData\Local\Microsoft\Windows\INetCache\Content.MSO\CABDB153.xlsx#'freq-exp4-rest-opt1'!A1) | 0.066 | 0.000 | 0.079 | <0.0001 | 251.490 | No |
| [Exponential 5 (CV - normal)](file:///C:\Users\tonge015\AppData\Local\Microsoft\Windows\INetCache\Content.MSO\CABDB153.xlsx#'freq-exp5-rest-opt1'!A1) | 0.066 | 0.000 | 0.079 | <0.0001 | 251.490 | No |
| [Hill (CV - normal)](file:///C:\Users\tonge015\AppData\Local\Microsoft\Windows\INetCache\Content.MSO\CABDB153.xlsx#'freq-hil-rest-opt1'!A1) | 0.008 | 0.007 | 0.010 | 0.985 | 188.287 | Yes |
| [Polynomial Degree 6 (CV - normal)](file:///C:\Users\tonge015\AppData\Local\Microsoft\Windows\INetCache\Content.MSO\CABDB153.xlsx#'freq-ply6-rest-opt1'!A1) | 0.914 | 0.677 | 2.935 | <0.0001 | 275.764 | No |
| [Polynomial Degree 5 (CV - normal)](file:///C:\Users\tonge015\AppData\Local\Microsoft\Windows\INetCache\Content.MSO\CABDB153.xlsx#'freq-ply5-rest-opt1'!A1) | 0.914 | 0.677 | 2.935 | <0.0001 | 275.764 | No |
| [Polynomial Degree 4 (CV - normal)](file:///C:\Users\tonge015\AppData\Local\Microsoft\Windows\INetCache\Content.MSO\CABDB153.xlsx#'freq-ply4-rest-opt1'!A1) | 0.914 | 0.677 | 2.935 | <0.0001 | 275.764 | No |
| [Polynomial Degree 3 (CV - normal)](file:///C:\Users\tonge015\AppData\Local\Microsoft\Windows\INetCache\Content.MSO\CABDB153.xlsx#'freq-ply3-rest-opt1'!A1) | 0.914 | 0.677 | 2.934 | <0.0001 | 275.764 | No |
| [Polynomial Degree 2 (CV - normal)](file:///C:\Users\tonge015\AppData\Local\Microsoft\Windows\INetCache\Content.MSO\CABDB153.xlsx#'freq-ply2-rest-opt1'!A1) | 0.914 | 0.677 | 2.935 | <0.0001 | 275.764 | No |
| [Power (CV - normal)](file:///C:\Users\tonge015\AppData\Local\Microsoft\Windows\INetCache\Content.MSO\CABDB153.xlsx#'freq-pow-rest-opt1'!A1) | 0.914 | 0.612 | 2.935 | <0.0001 | 275.764 | No |
| [Linear (CV - normal)](file:///C:\Users\tonge015\AppData\Local\Microsoft\Windows\INetCache\Content.MSO\CABDB153.xlsx#'freq-lin-unrest-opt1'!A1) | 0.914 | 0.612 | 2.935 | <0.0001 | 275.764 | Yes |

### Supplementary material S3.2. BMD modelling of the predicted anti-androgenic response of FLU +HF

**Supplementary Table S1.3.2.1.** Input values of the predicted dose-response data of FLU +HF.

| **Dose (mg/kg)** | **n** | **response** | **SD** |
| --- | --- | --- | --- |
| 0.002 | 3.00 | 100.00 | 0.00 |
| 0.008 | 3.00 | 100.47 | 3.61 |
| 0.02 | 3.00 | 85.84 | 14.98 |
| 0.08 | 3.00 | 56.06 | 15.64 |
| 0.23 | 3.00 | 24.46 | 8.03 |
| 0.78 | 3.00 | 8.18 | 4.42 |
| 2.33 | 3.00 | 2.78 | 1.37 |

**Supplementary Table S1.3.2.2.** BMD analysis of the predicted dose-response data of FLU +HF. BMD_05_, BMDL_05,_ and BMDU_05_ values were obtained using BMDS software version 3.2.1, at a BMD of 5% extra risk, BMR type Relative Deviation with normal distribution and constant variance.

| Model | BMD (mg/kg) | BMDL (mg/kg) | BMDU (mg/kg) | Test 4 P‑Value | AIC | Accepted |
| --- | --- | --- | --- | --- | --- | --- |
| [Exponential 2 (CV - normal)](file:///C:\Users\tonge015\AppData\Local\Microsoft\Windows\INetCache\Content.MSO\CABDB153.xlsx#'freq-exp2-rest-opt1'!A1) | 8.81 | 6.95 | 12.17 | <0.0001 | 251.49 | No |
| [Exponential 3 (CV - normal)](file:///C:\Users\tonge015\AppData\Local\Microsoft\Windows\INetCache\Content.MSO\CABDB153.xlsx#'freq-exp3-rest-opt1'!A1) | 8.81 | 6.95 | 22.50 | <0.0001 | 251.49 | No |
| [Exponential 4 (CV - normal)](file:///C:\Users\tonge015\AppData\Local\Microsoft\Windows\INetCache\Content.MSO\CABDB153.xlsx#'freq-exp4-rest-opt1'!A1) | 8.81 | 6.95 | 12.17 | <0.0001 | 251.49 | No |
| [Exponential 5 (CV - normal)](file:///C:\Users\tonge015\AppData\Local\Microsoft\Windows\INetCache\Content.MSO\CABDB153.xlsx#'freq-exp5-rest-opt1'!A1) | 8.81 | 6.95 | 22.50 | <0.0001 | 251.49 | No |
| [Hill (CV - normal)](file:///C:\Users\tonge015\AppData\Local\Microsoft\Windows\INetCache\Content.MSO\CABDB153.xlsx#'freq-hil-rest-opt1'!A1) | 0.39 | 0.11 | 1.20 | 0.98 | 188.29 | Yes |
| [Polynomial Degree 6 (CV - normal)](file:///C:\Users\tonge015\AppData\Local\Microsoft\Windows\INetCache\Content.MSO\CABDB153.xlsx#'freq-ply6-rest-opt1'!A1) | 269.25 | 170.66 | 1032.61 | <0.0001 | 275.76 | No |
| [Polynomial Degree 5 (CV - normal)](file:///C:\Users\tonge015\AppData\Local\Microsoft\Windows\INetCache\Content.MSO\CABDB153.xlsx#'freq-ply5-rest-opt1'!A1) | 269.25 | 170.90 | 1032.74 | <0.0001 | 275.76 | No |
| [Polynomial Degree 4 (CV - normal)](file:///C:\Users\tonge015\AppData\Local\Microsoft\Windows\INetCache\Content.MSO\CABDB153.xlsx#'freq-ply4-rest-opt1'!A1) | 269.25 | 170.73 | 1032.61 | <0.0001 | 275.76 | No |
| [Polynomial Degree 3 (CV - normal)](file:///C:\Users\tonge015\AppData\Local\Microsoft\Windows\INetCache\Content.MSO\CABDB153.xlsx#'freq-ply3-rest-opt1'!A1) | 269.25 | 170.73 | 1032.61 | <0.0001 | 275.76 | No |
| [Polynomial Degree 2 (CV - normal)](file:///C:\Users\tonge015\AppData\Local\Microsoft\Windows\INetCache\Content.MSO\CABDB153.xlsx#'freq-ply2-rest-opt1'!A1) | 269.25 | 170.67 | 1032.61 | <0.0001 | 275.76 | No |
| [Power (CV - normal)](file:///C:\Users\tonge015\AppData\Local\Microsoft\Windows\INetCache\Content.MSO\CABDB153.xlsx#'freq-pow-rest-opt1'!A1) | 269.25 | 170.67 | 1032.61 | <0.0001 | 275.76 | No |
| [Linear (CV - normal)](file:///C:\Users\tonge015\AppData\Local\Microsoft\Windows\INetCache\Content.MSO\CABDB153.xlsx#'freq-lin-unrest-opt1'!A1) | 269.25 | 170.66 | 1032.61 | <0.0001 | 275.76 | Yes |

**
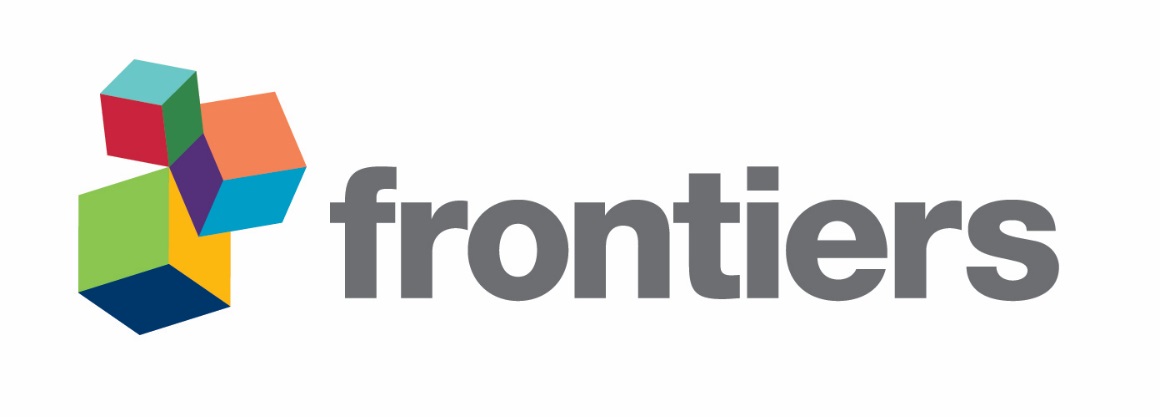
**
